# *De Novo* Genome Sequence of *Yersinia aleksiciae* Y159^T^

**DOI:** 10.1128/genomeA.01066-15

**Published:** 2015-09-17

**Authors:** Lisa D. Sprague, Heinrich Neubauer

**Affiliations:** Friedrich-Loeffler-Institut, Federal Research Institute for Animal Health, Institute of Bacterial Infections and Zoonoses, Jena, Germany

## Abstract

We report here on the genome sequence of *Yersinia aleksiciae* Y159^T^, isolated in Finland in 1981. The genome has a size of 4 Mb, a G+C content of 49%, and is predicted to contain 3,423 coding sequences.

## GENOME ANNOUNCEMENT

Originally typed as *Yersinia kristensenii*, *Yersinia aleksiciae* was identified as a separate species on the basis of differences in DNA-DNA relatedness, the presence of a specific 16S rRNA gene sequence, and the existence of lysine decarboxylase activity (1). *Y. aleksiciae* lack the plasmid-borne markers for pathogenicity and are considered to be apathogenic. *Y. aleksiciae* strains have been isolated from reindeer, rat, mole, pig, and human feces. *Y. aleksiciae* appears not to be host specific and might be part of the physiological intestinal flora in mammals. *Y. aleksiciae* Y159^T^ was isolated from human feces in 1981 in Finland and belongs to serogroup O:16 (1).

This paper announces the *de novo* genome sequence of *Y. aleksiciae* Y159^T^. Genomic DNA was isolated from an overnight culture grown in LB medium with 1% glucose with a Qiagen genomic tip 100/Q and the genomic DNA buffer set (Qiagen, Hilden, Germany). DNA quality was examined by using both a NanoDrop spectrophotometer (Thermo Scientific, Schwerte, Germany) and a Qubit 2.0 fluorometer (Life Technologies, Darmstadt, Germany). Whole-genome sequencing was performed on the Pacific Biosciences RS sequencer with SMRT Technology PacBio RS II (Pacific Biosciences, Menlo Park, CA, USA), at GATC Biotech (Konstanz, Germany), using standard protocols according to the manufacturer’s instructions, which were followed throughout the sequencing process. *De novo* assembly was performed with SMRT portal version 2.1.0 (Daemon version 2.1.0, SMRT View version 2.1, and SMRTpipe version 2.1.0; Pacific Biosciences) from eight contigs, with an *N*_50_ contig length of 3,099,996 bp. The genome has a size of 4,000,307 bp and a G + C content of 49% (Geneious 8.1.3; Biomatters, Auckland, New Zealand). Genome annotation was done using the NCBI Prokaryotic Genome Annotation Pipeline (http://www.ncbi.nlm.nih.gov/genome/annotation_prok/) and predicted 3,576 genes, 57 pseudogenes, 3,423 coding sequences (CDSs), 73 tRNAs, 22 rRNA genes, and 1 noncoding RNA (ncRNA) gene.

### Nucleotide sequence accession number.

The sequence of *Y. aleksiciae* Y159^T^ has been deposited at DDBJ/EMBL/Genbank under the accession no. CP011975.
